# Association of Plasma BDNF Concentration and Val66Met Polymorphism with Postoperative Delirium After Cardiac Surgery Under General Anesthesia with Cardiopulmonary Bypass

**DOI:** 10.3390/jcm14217690

**Published:** 2025-10-29

**Authors:** Kacper Lechowicz, Aleksandra Szylińska, Elżbieta Cecerska-Heryć, Ewa Ostrycharz-Jasek, Edyta Zagrodnik, Jerzy Pacholewicz, Barbara Dołęgowska, Katarzyna Kotfis

**Affiliations:** 1 Department of Anesthesiology, Intensive Care and Pain Management, Pomeranian Medical University, Al. Powstańców Wielkopolskich 72, 70-111 Szczecin, Poland; kacper.lechowicz@pum.edu.pl (K.L.); katarzyna.kotfis@pum.edu.pl (K.K.); 2 Department of Cardiac Surgery, Pomeranian Medical University, Al. Powstańców Wielkopolskich 72, 70-111 Szczecin, Poland; jerzy.pacholewicz@pum.edu.pl; 3 Department of Laboratory Medicine, Pomeranian Medical University, Al. Powstańców Wielkopolskich 72, 70-111 Szczecin, Poland; elzbieta.cecerska.heryc@pum.edu.pl (E.C.-H.); barbara.dolegowska@pum.edu.pl (B.D.); 4 Department of Medical Analytics, Pomeranian Medical University, Al. Powstańców Wielkopolskich 72, 70-111 Szczecin, Poland; ewa.ostrycharz@pum.edu.pl; 5 Clinical Department of Anesthesiology and Intensive Care of Adults and Children, Pomeranian Medical University in Szczecin, 71-460 Szczecin, Poland; edyta.zagrodnik@pum.edu.pl

**Keywords:** brain-derived neurotrophic factor (BDNF), Val66Met polymorphism, postoperative delirium, cardiac surgery, cardiopulmonary bypass, general anesthesia, neurocognitive dysfunction

## Abstract

**Background/Objectives:** Cardiac surgery, particularly procedures performed with cardiopulmonary bypass (CPB), carries a high risk of neurological complications, including postoperative delirium (POD), which affects 16–73% of patients and increases the likelihood of long-term cognitive impairment. Brain-derived neurotrophic factor (BDNF), a neurotrophin involved in neuronal function, synaptic plasticity, and inflammatory regulation processes, including its Val66Met polymorphism, has been implicated as a potential predictor of POD. This study aimed to evaluate the relationship between perioperative plasma BDNF levels, the BDNF Val66Met polymorphism, and the incidence of POD in patients undergoing elective cardiac surgery with CPB. **Methods:** This prospective observational single-center study enrolled 287 adults scheduled for elective isolated coronary artery bypass grafting (CABG) with CPB, of whom 107 met all inclusion criteria for final analysis. Exclusion criteria included urgent surgery and pre-existing cognitive or psychiatric disorders. Preoperative evaluation included cognitive testing (MoCA), laboratory and biochemical analysis, and genotyping for BDNF Val66Met. Postoperatively, patients were assessed for POD using the CAM-ICU scale for the first three consecutive days. Cognitive function (using MoCA) and other neurological complications were evaluated during hospitalization, at 30-day and 12-month follow-up. Associations between biomarkers, genetic factors, and clinical outcomes were analyzed. **Results:** POD occurred in 19.6% of patients who were older, had higher EuroSCORE II, greater coronary disease burden, more frequent prior stroke and chronic kidney disease, and lower neutrophil counts. POD was significantly associated with prolonged hospital stay, need for continuous renal replacement therapy, and reoperation. The BDNF Val66Met polymorphism was present in 31.8% of patients but was not associated with POD, although carriers exhibited higher plasma BDNF concentrations across all time points. **Conclusions:** Perioperative plasma BDNF concentrations and the BDNF Val66Met polymorphism were not independently associated with the occurrence of POD in elective CABG patients. However, POD was significantly linked to prolonged hospitalization and reoperations. Neurological complications remain an important challenge in cardiac surgery, emphasizing the need for further research and early identification strategies to improve postoperative outcomes.

## 1. Introduction

Each year, more than two million cardiac surgical procedures are performed worldwide, with an increasing proportion of patients being elderly individuals with comorbidities such as hypertension and diabetes mellitus. This patient population is at heightened risk of postoperative complications, including neurological sequelae [1]. According to the American College of Cardiology (ACC) and the American Heart Association (AHA) classification, neurological complications following cardiac surgery are categorized as type I neuronal injuries (e.g., stroke, transient ischemic attacks) and type II injuries, which encompass postoperative delirium and postoperative cognitive dysfunction (POCD) [2].

Delirium is an acute disturbance of consciousness and attention, accompanied by alterations in cognitive processes such as memory, orientation, and perception [3]. It typically develops within hours to days and is characterized by a fluctuating course of symptoms throughout the day. Delirium is classified according to its etiology—for example, alcohol-related delirium or delirium secondary to critical illness, with sedative-associated, hypoxic, and septic delirium and unclassified phenotypes [4]. In surgical patients, the condition is referred to as postoperative delirium (POD), although diagnostic criteria remain identical to those used for delirium of other causes [3,5]. Management is clearly defined in the updated POD guidelines of the European Society of Anaesthesiology and Intensive Care (ESAIC) [6].

Three clinical subtypes of delirium are recognized: hyperactive, hypoactive, and mixed. This classification is based primarily on psychomotor features [3,7,8,9]. Hyperactive delirium is characterized by increased psychomotor agitation, restlessness, aggression, and frequent hallucinations or delusions [10,11]. In contrast, hypoactive delirium is marked by social withdrawal, apathy, excessive somnolence, and reduced responsiveness to external stimuli. Due to its subtle clinical presentation and absence of overt agitation, hypoactive delirium frequently remains underdiagnosed, contributing to poorer outcomes and increased risk of long-term health consequences [7,8,9].

Despite these differences, all delirium subtypes share certain core clinical features. The most prominent are disturbances of attention and awareness, manifesting as difficulty focusing, inability to follow instructions, and disorientation regarding time, place, or even personal identity [8,12]. Sleep–wake cycle disruptions are also common, with nocturnal insomnia and excessive daytime somnolence impairing patient functioning and presenting further clinical challenges [13,14]. Persistent delirium, lasting for weeks or months, is not uncommon, and approximately 20% of patients still exhibit symptoms at six months post-onset [15].

Accurate recognition of delirium is essential for timely interventions that reduce stress, improve comfort, and shorten its duration [3,16,17]. Education of healthcare providers in recognizing delirium subtypes enables earlier management and helps prevent adverse outcomes [7,9,16,18,19,20].

Brain-derived neurotrophic factor (BDNF) is a neurotrophin mainly expressed in the central nervous system, which regulates neuronal survival, differentiation, and synaptic plasticity [21,22]. It is critical for neurogenesis, neuronal regeneration, and cellular homeostasis, while also modulating brain plasticity in response to environmental stimuli. Beyond neurodevelopment, BDNF has been linked to immunological processes and cognitive regulation [22,23,24].

Cardiac surgery with cardiopulmonary bypass induces significant physiological and inflammatory stress that may alter circulating and cerebral BDNF concentration. Reduced BDNF concentrations have been associated with impaired neuronal recovery and increased susceptibility to neurocognitive disorders, including delirium [5,25,26].

A common genetic variant of the BDNF gene, Val66Met, results from a valine-to-methionine substitution at codon 66, affecting protein processing and secretion. Epidemiological studies suggest that the Met allele occurs in the global population with a frequency ranging from approximately 20% to as much as 50% in selected ethnic groups, although specific values depend on geographic and ethnic origin. In the European population, the frequency of this mutation (heterozygous or homozygous) is estimated at approximately 15–30%, making it the most common mutation in this gene [27,28]. This polymorphism has been implicated in psychiatric and neurodegenerative conditions, and carriers of the Met allele may be more vulnerable to memory disturbances and neuroinflammatory responses under systemic stress [22,28,29,30,31,32]. These findings suggest that BDNF Val66Met may represent a genetic determinant of susceptibility to postoperative delirium [32,33].

### Aim of the Study

The aim of this study is to investigate the impact of circulating BDNF concentrations and the BDNF Val66Met polymorphism on the occurrence of postoperative delirium in patients undergoing cardiac surgery with cardiopulmonary bypass.

## 2. Materials and Methods

A prospective observational clinical study was conducted between September 2020 and June 2024 at a tertiary university hospital of the Pomeranian Medical University in Szczecin.

### 2.1. Ethical Considerations

The study was conducted according to the guidelines of the Declaration of Helsinki and approved by the Institutional Review Board, i.e., Bioethics Committee of the Pomeranian Medical University in Szczecin, approval no. KB-0012/55/19 issued on 25 February 2019. All eligible patients who agreed to participate received detailed information about the study from a member of the research team and signed written informed consent before enrolment and prior to the initiation of study procedures. To ensure data confidentiality, all analyses were performed on coded (anonymized) data.

### 2.2. Patient Enrollment

Patients enrolled in this prospective observational study were hospitalized in the Department of Cardiac Surgery in the University Hospital No. 2 of the Pomeranian Medical University. Under a strict study protocol, we enrolled only patients undergoing elective isolated coronary artery bypass grafting (CABG) with the use of cardiopulmonary bypass to ensure a homogeneous study population. Postoperatively, patients were admitted to the post-anaesthesia care unit (PACU) and later returned to the ward at the Department of Cardiac Surgery.

Each enrolled patient received complete information regarding the study from a research team member and was given the opportunity to ask questions and obtain clarifications, in accordance with ICH-GCP standards. Only those patients who signed informed consent for participation, follow-up visits, and both direct and indirect contact during follow-up were included in the study.

### 2.3. Inclusion Criteria

According to the study protocol, patients were eligible for enrolment if they met the following criteria:

1. Male or female, aged 18–90 years.
2. Scheduled for elective CABG surgery with the use of cardiopulmonary bypass.
3. Provided written informed consent in accordance with ICH-GCP and national regulations.

### 2.4. Exclusion Criteria

Patients were excluded from the study if any of the following criteria applied:

1. Emergency surgical procedures.
2. Patients initially qualified for CABG, in whom the surgical approach was modified intraoperatively.
3. History of cognitive impairment.
4. History of psychiatric disorders (including depression, dementia, psychosis, or schizophrenia).

The study included preoperative, perioperative, and postoperative assessments of each participant.

### 2.5. Demographic Data and Baseline Assessment

All data were collected through patient interviews and/or review of medical records. Information was recorded using study-specific case report forms (CRFs). In addition to demographic characteristics, attention was given to comorbidities and chronic medication use. Preoperatively, cognitive function was assessed using the Montreal Cognitive Assessment (MoCA, © Z. Nasreddine, MoCA, Montreal, QC, Canada). Baseline data also included medical history, physical examination, and standard anthropometric measurements. The preoperative evaluation comprised routine laboratory tests, including complete blood count, inflammatory markers, renal function parameters, glycated haemoglobin, and arterial blood gas analysis.

### 2.6. Perioperative Period

All patients underwent elective cardiac surgery using cardiopulmonary bypass under general anesthesia with endotracheal intubation, following a standardised institutional anesthesia protocol. Induction of general anesthesia was achieved using propofol (Fresenius Kabi, Bad Homburg, Germany) and fentanyl (Polfa Warszawa S.A., Warsaw, Poland), followed by neuromuscular blockade with pancuronium (Jelfa S.A., Jelenia Góra, Poland). Endotracheal intubation was performed, and anesthesia was subsequently maintained with inhaled sevoflurane (AbbVie Inc., North Chicago, IL, USA).

Intraoperative analgesia was provided with fentanyl (Polfa Warszawa S.A., Warsaw, Poland). Prior to surgery, patients provided additional consent for genetic testing. During surgery, blood samples were collected at three different time points for BDNF concentration analysis.

### 2.7. Postoperative Period

The postoperative period was monitored in accordance with the standard institutional protocol for patient management and surveillance. In the immediate postoperative phase, patients were assessed for central nervous system dysfunction. Agitation was evaluated using the Richmond Agitation-Sedation Scale (RASS), while delirium was screened with the Confusion Assessment Method for the Intensive Care Unit (CAM-ICU), performed twice daily—between 6:00–8:00 a.m. and 4:00–6:00 p.m. for the first 3 postoperative days. A structured neurological examination was also performed to detect focal neurological deficits, transient ischemic attack (TIA), stroke, or motor weakness.

Between 48 and 72 h after surgery, the MoCA was repeated to assess changes in cognitive performance. For the first three postoperative days, patients were assessed twice daily with the CAM-ICU to confirm or exclude delirium, alongside evaluation of pain intensity using the Numeric Rating Scale (NRS). The management for POD and pain followed a standardised institutional protocol for all patients undergoing cardiac surgery and included both diagnostic procedures and non-pharmacological interventions, as well as pharmacological treatment (dexmedetomidine, haloperidol) if needed. These assessments were repeated one month after surgery, coinciding with the routine postoperative follow-up visit.

### 2.8. Long-Term Follow-Up

All patients were followed for 12 months postoperatively. Cognitive function was reassessed using the MoCA, and persistent postoperative pain was evaluated. During the one-year follow-up, the incidence of neurological complications was recorded, including ischemic stroke, reversible ischemic neurologic deficit (RIND), transient ischemic attack (TIA), postoperative delirium (POD), ICU delirium (severe delirium), seizures, postoperative depression, persistent postoperative pain, and postoperative cognitive dysfunction (POCD).

### 2.9. Genetic Analysis

Blood samples collected for genetic analysis were subjected to DNA isolation using Genomic Mini AX Blood 300 Spin kit (A&A Biotechnology, Gdańsk, Poland) and amplification using Real-Time PCR System (Thermo Fisher Scientific, Waltham, MA, USA). The genetic assessment included evaluation of the frequency of the BDNF Val66Met polymorphism (G196A, rs6265, located at 11p13), with classification of patients according to homozygous and heterozygous variants, using PCR Mix Plus Green kit (A&A Biotechnology, Gdańsk, Poland). A standardized genetic test was used only for this single gene.

### 2.10. Biochemical Analysis

Serum BDNF concentrations were measured using the Human BDNF ELISA Kit (Sigma-Aldrich, St. Louis, MO, USA) according to the manufacturer’s instructions. Absorbance was measured using the EnVision 2104 (PerkinElmer, Waltham, MA, USA), and results were expressed in pg/mL and corrected for haematocrit.

### 2.11. Statistical Analysis

The study population was stratified into two subgroups based on the presence or absence of delirium (DEL+ and DEL−). Epidemiological and clinical data, leukocyte indices, and biochemical and genetic results were analyzed in relation to the following outcomes: ICU mortality, in-hospital mortality, duration of mechanical ventilation, incidence of neurological complications (including stroke, transient ischemic attack, postoperative seizures, severe delirium, frequency and severity of cognitive impairment, and severity of postoperative pain).

All statistical analyses were performed using Statistica 13 software (StatSoft, Inc., Tulsa, OK, USA). Median and interquartile ranges (upper and lower quartiles) were calculated for continuous variables, whereas nominal and categorical data were presented as percentages. Since the distribution of continuous variables deviated from normality, non-parametric tests (Mann–Whitney U test) were applied.

The chi-square test was used for nominal data, while within-group comparisons were performed using the Wilcoxon test. Logistic regression analysis was employed to assess associations between variables. A *p*-value < 0.05 was considered statistically significant.

Graphical presentation

Figures depicting plasma BDNF concentrations were generated using median and interquartile range (IQR, Q1–Q3) values derived directly from the summary data in corresponding tables. Box-and-whisker plots display the interquartile range as the box, the median as a horizontal line, and whiskers limited to the IQR. Distinct colors were applied to differentiate study groups for clarity and consistency.

## 3. Results

### 3.1. Study Population

Of the 568 patients initially screened, 281 were excluded due to not meeting the inclusion criteria (urgent or complex surgical procedures). Among the 287 patients eligible for intraoperative enrolment, 179 were excluded intraoperatively: in 144 cases due to modification of the planned surgical procedure, and in 35 cases for perioperative reasons determined by the study team (including failure to collect blood samples, haemolysis, or incomplete neurocognitive testing within protocol-defined time windows).

**Figure 1:**
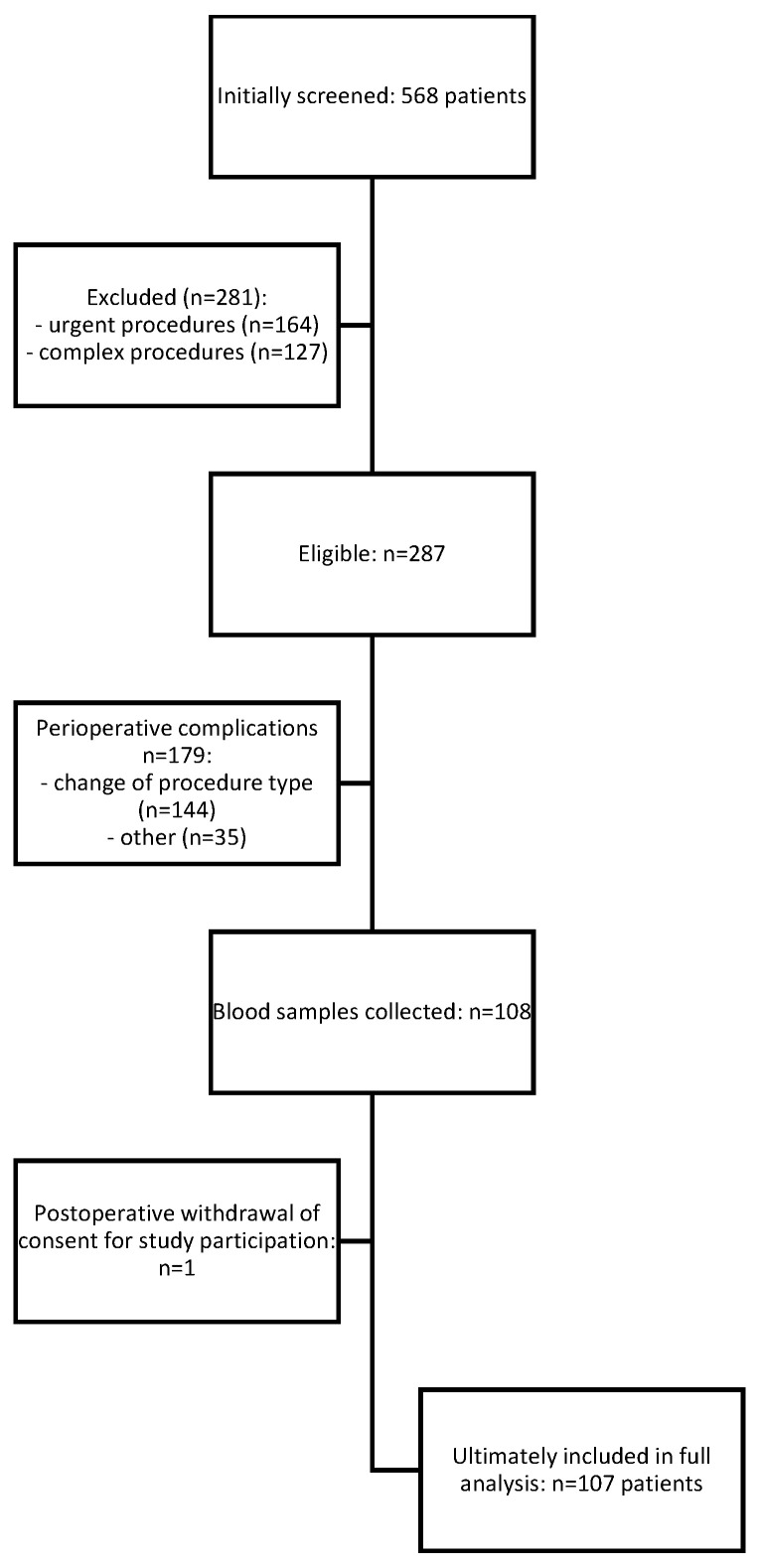
Study protocol—STROBE diagram of the study.

Of the remaining 108 patients, one withdrew consent during follow-up. Thus, 107 patients were included in the final analysis (Figure 1). The primary stratification criterion was the presence or absence of postoperative delirium (POD) during hospitalization.

Baseline characteristics of the study cohort are summarized in Table 1 and Table 2. The mean age was 67 years, and most patients were overweight. Median left ventricular ejection fraction was 45%.

The majority were men (82.6%), and the most prevalent comorbidities were hypertension, hypercholesterolemia, and prior myocardial infarction.

### 3.2. Preoperative Characteristics

Patients were divided into those who experienced POD (DEL+) and who did not experience POD (DEL−) groups. Demographic and comorbidity data are shown in Table 3 and Table 4. The proportion of men and women did not differ significantly between groups. Hypertension, hypercholesterolemia, and prior myocardial infarction were the most common comorbidities overall.

There were no significant demographic differences between groups except for age, which was higher in the DEL+ group (69 vs. 67 years, *p* = 0.031). Patients with POD exhibited more advanced coronary artery disease, as measured by the CCS (Canadian Cardiovascular Society) angina grading scale (*p* = 0.006), and had higher predicted operative risk according to EuroSCORE II (median 3.54 vs. 2.25, *p* = 0.006). Prior stroke and chronic kidney disease were also significantly associated with POD.

Notably, POD was more frequent in patients receiving low-molecular-weight heparin before surgery, although not as bridging therapy during anticoagulant switching. No associations were found between POD and other preoperative medications.

There were no significant group differences in BMI, frailty score, baseline left ventricular ejection fraction, or ASA physical status. Similarly, the prevalence of other comorbidities (including ischemic heart disease, chronic heart failure, atrial fibrillation, diabetes, peripheral vascular disease, thyroid disease, COPD, cancer, or autoimmune disorders) did not differ between groups.

Preoperative laboratory testing revealed significantly lower baseline neutrophil counts in the DEL+ group (*p* = 0.032). Other baseline laboratory values were comparable between groups (Table 5).

### 3.3. Intraoperative Findings

Intraoperative data are presented in Table 6 and Table 7. The pre-, intra-, and post-bypass plasma concentrations of brain-derived neurotrophic factor (BDNF) were lower in the DEL+ group, though differences did not reach statistical significance. Duration of anesthesia, cardiopulmonary bypass (CPB) time, total surgical time, and the type of cardioplegia used were not associated with POD incidence. Similarly, transfusion of blood products in the perioperative or early postoperative period did not influence POD risk.

**Figure 2:**
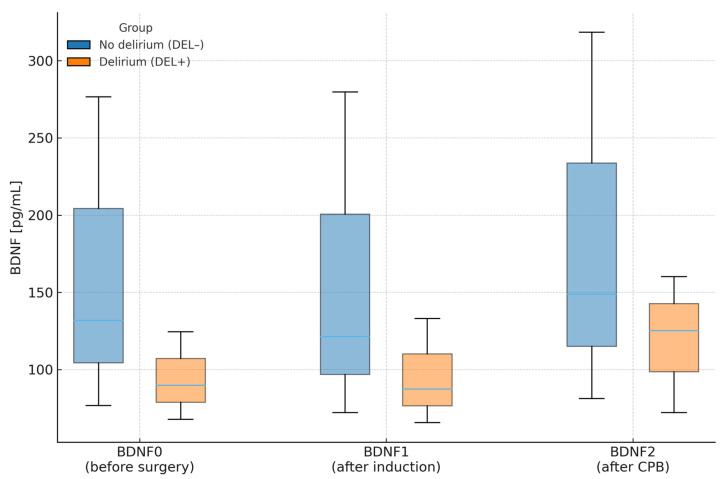
Plasma BDNF concentrations (median and interquartile range) at three perioperative time points in patients with (DEL+) and without (DEL−) postoperative delirium. Legend: Boxes show IQR (Q1–Q3); horizontal line = median. CPB—cardiopulmonary bypass.

Figure 2 illustrates the median and IQR BDNF concentrations across perioperative time points in patients with (DEL+) and without (DEL−) delirium, corresponding directly to data presented in Table 6. Consistent with those results, patients who developed delirium tended to have lower perioperative BDNF levels, though these differences were not statistically significant. Perioperative trends within each group followed similar patterns without meaningful temporal variation.

### 3.4. Postoperative Outcomes

As shown in Table 8, postoperative acute kidney injury requiring renal replacement therapy was strongly associated with POD (*p* < 0.001), as was the need for reoperation (*p* = 0.036). POD was also associated with prolonged length of stay (median 8 vs. 6 days, *p* = 0.012). In contrast, POD did not significantly affect postoperative mortality.

### 3.5. Cognitive Assessment

Cognitive function was assessed perioperatively using the Richmond Agitation-Sedation Scale (RASS) and the Montreal Cognitive Assessment (MoCA). For statistical analysis, MoCA scores were categorized into four groups: 0 (no impairment), 1 (mild impairment), 2 (moderate impairment), and 3 (severe impairment). Postoperative testing (48–72 h) revealed a strong correlation between POD occurrence and impaired MoCA performance, consistent with the cognitive deficits inherent to delirium (Table 8).

### 3.6. BDNF Val66Met Polymorphism

Genotyping for the BDNF Val66Met polymorphism was performed in all 107 patients (Table 9). The variant was present in 34 patients (31.8%), including 31 heterozygotes and 3 homozygotes. The distribution of the polymorphism did not differ significantly between groups. Interestingly, the variant appeared more frequently in patients without POD (34.9% vs. 19.1%), though this trend did not reach statistical significance (*p* = 0.126).

**Figure 3:**
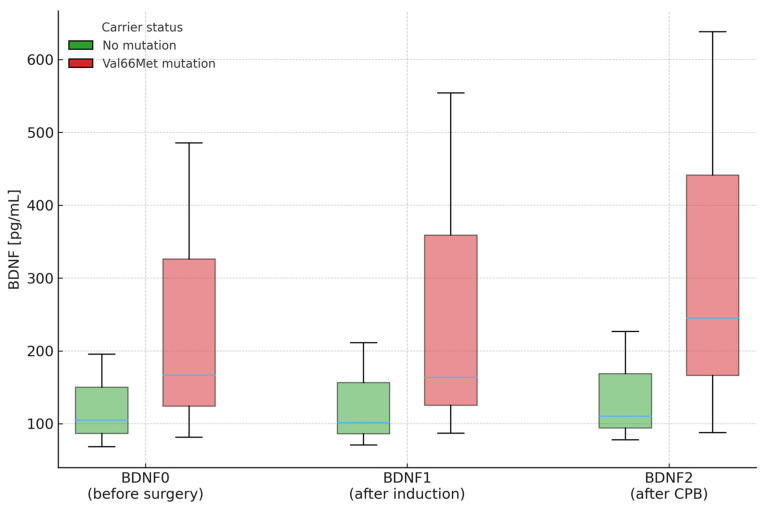
Plasma BDNF concentrations (median and interquartile range) at three perioperative time points stratified by Val66Met carrier status. Legend: Labels as above. Boxes show IQR (Q1–Q3); horizontal line = median. CPB—cardiopulmonary bypass.

Across all perioperative time points, carriers of Val66Met exhibited higher plasma BDNF concentrations than non-carriers (BDNF0 = 166.48 vs. 104.73 pg/mL, *p* = 0.003; BDNF1 = 163.68 vs. 101.56 pg/mL, *p* = 0.024; BDNF2 = 244.89 vs. 110.24 pg/mL, *p* = 0.010), although within-patient changes (ΔBDNF) did not differ significantly between groups. Logistic-regression analysis confirmed a significant association between the polymorphism and absolute BDNF concentrations at early perioperative time points. Figure 3 depicts these medians and interquartile ranges exactly as reported in Table 9.

### 3.7. Logistic Regression of Perioperative Risk Factors for POD

Multivariable logistic regression was used to assess the association between perioperative factors and POD (Table 10). POD was independently associated with prolonged hospital stay (OR = 1.204, *p* = 0.009), reoperation (OR = 6.510, *p* = 0.021), and renal replacement therapy (OR = 26.563, *p* = 0.004), as visible in Table 10. Preoperative C-reactive protein (CRP) concentration was also an independent predictor of POD (OR = 1.187, *p* = 0.039).

## 4. Discussion

This study examined the relationship between perioperative BDNF concentrations, the Val66Met polymorphism, and postoperative delirium (POD) incidence in patients undergoing isolated coronary artery bypass grafting (CABG). Our key findings demonstrate that while neither BDNF levels nor the Val66Met polymorphism independently predicted POD, delirium occurred in 19.6% of patients and was strongly associated with adverse postoperative outcomes, including prolonged hospitalization, reoperations, and renal complications. These results contribute to the evolving understanding of perioperative neurocognitive disorders and their clinical implications.

The study cohort represented a typical population undergoing CABG in our institution, with demographic and clinical characteristics consistent with other reports of cardiac surgical patients [19,34,35]. The mean age was 67 years, and the majority were men. The prototypical participant was overweight (BMI 29.4), hypertensive, hypercholesterolemic, and had a history of myocardial infarction. These preoperative comorbidities reflect the well-established continuum of cardiovascular risk factors observed in cardiac surgical populations worldwide [12,36,37,38]. Interestingly, only 14% of patients had a documented history of peripheral vascular disease, and despite a high prevalence of smoking, the proportion with a diagnosis of chronic obstructive pulmonary disease (COPD) was low (4.7%). This may reflect underdiagnosis rather than true absence of disease.

Santos et al. studied a Brazilian cohort and reported a somewhat different risk factor profile compared with our population. In their study, hypertension (>70%) and diabetes were the most prevalent comorbidities, while smoking was less common (~45%), and overweight status was present in <10% of patients. Beta-blockers, nitrates, and diuretics were most frequently used, whereas statin use was not reported [39]. Given the study’s date (2004), both therapeutic strategies and socioeconomic conditions have changed substantially, which may account for these differences. Similarly, Yokoyama et al., analysing Japanese cardiac surgery patients, reported hypertension (45%) and diabetes (40%) as leading comorbidities [40].

In our cohort, no patients experienced stroke or transient ischemic attack (TIA) during hospitalization, while POD occurred in 21 patients (19.6%). The reported incidence of neurological complications after cardiac surgery varies across studies. Raffa et al., in a retrospective analysis of 2121 patients undergoing various cardiac procedures, found major stroke in 1.7% and TIA in 2.5% [41]. Roach et al. (1996) reported a 2.8% incidence of perioperative myocardial infarction and 2.7% incidence of cognitive impairment following elective CABG [42]. Bruggemans et al., in a prospective study of 101 CABG patients, demonstrated memory impairment in 64% at discharge, with nearly complete resolution by 3 months [43].

POD remains a serious and independent complication of postoperative care in cardiac surgery [44]. Patients who developed POD were, on average, nearly 4 years older (70.1 vs. 66.3 years). This age effect is well recognized, with large-scale studies consistently identifying advanced age as a robust risk factor for POD [15,45]. Advanced age is not only a determinant of delirium after surgery, but also across general hospitalized and critically ill populations, with prevalence reported to exceed 70% in some ICU cohorts [46,47]. Standardized diagnostic tools such as CAM-ICU or ICDSC are therefore recommended, and their routine use is part of our institutional practice [8]. The most recent ESAIC guidelines recommend incorporating age, ASA physical status, comorbidities, and cognitive screening tools such as MMSE or MoCA into POD risk assessment [6].

In our study, a high proportion of patients reported regular or occasional alcohol consumption, and only 28% were nonsmokers. Moreover, POD incidence was associated with severity of coronary artery disease (measured by CCS classification), history of stroke, and chronic kidney disease. These findings align with prior studies [48,49]. Gosselt et al. demonstrated associations between POD, age, and renal dysfunction, though unlike our findings and prior work from our center, they did not observe a significant impact of coronary artery disease severity [49,50,51].

### 4.1. Preoperative Laboratory Parameters

Patients who developed delirium exhibited lower preoperative neutrophil counts, accompanied by a trend toward lower leukocyte and haemoglobin levels and higher creatinine and CRP. Although none of these parameters exceeded laboratory reference ranges or reached consistent statistical significance, the directionality is noteworthy. Previous studies have identified inflammatory and hematologic derangements as potential contributors to POD. Santos et al. reported higher preoperative urea and creatinine levels in delirium patients, while Rudolph et al. described similar associations, albeit without statistical robustness [38,39]. Our findings suggest that subtle immune or hematologic alterations may lower physiological reserve, thereby predisposing patients to postoperative cognitive vulnerability.

### 4.2. Anticoagulant Therapy

A noteworthy observation was the association between low-molecular-weight heparin (LMWH) use and increased POD incidence. This represents a novel finding in cardiac surgery literature, as previous studies have not systematically examined this relationship. While we interpret this primarily as confounding by indication—patients receiving LMWH typically have greater comorbidity burden—the association warrants further investigation. Interestingly, evidence from non-surgical populations suggests LMWH may have neuroprotective properties, highlighting the need for cardiac surgery-specific research into anticoagulant strategies and their neurocognitive implications [39].

### 4.3. Cardiopulmonary Bypass and Operative Factors

Contrary to expectations, intraoperative variables—including duration of anesthesia, total operative time, and CPB duration—did not significantly differ between delirium and non-delirium patients. Although patients with POD had slightly longer CPB times, the difference was not statistically significant. These results are consistent with those of Rudolph et al., who observed trends toward prolonged CPB and anesthetic exposure in delirium patients but failed to demonstrate independent predictive value [38]. However, the biological plausibility of CPB-related neuroinflammation and microembolization as contributors to POD remains valid and deserves continued mechanistic investigation.

The conducted study showed that patients who developed postoperative delirium received more units of packed red blood cells transfusions than patients without delirium. The result did not achieve statistical significance. This is consistent with data reported in the literature. Gosselt et al. demonstrated that transfusion of blood products is one of the most significant risk factors for delirium after procedures using cardiopulmonary bypass [50].

### 4.4. Postoperative Complications

Delirium was strongly associated with adverse postoperative trajectories. Patients with POD required reoperation and renal replacement therapy more frequently and experienced prolonged hospitalization. Importantly, AKI and the need for dialysis emerged as robust correlates of delirium, consistent with prior reports linking renal dysfunction to acute cerebral complications. Siew et al. demonstrated that AKI independently predicts delirium and coma in critically ill patients, while Kotfis et al. reported a tenfold increased risk of POD following CABG in patients with AKI [51,52]. In contrast to some multicenter series, we did not observe increased mortality among delirium patients, possibly reflecting sample size limitations or the effectiveness of early detection and management strategies within our ICU setting.

### 4.5. Dynamics of BDNF Levels

BDNF is increasingly recognized as a critical mediator of synaptic plasticity, neuroinflammation, and neuronal survival [53]. In our study, pre-, intra-, and post-bypass plasma concentrations of brain-derived neurotrophic factor (BDNF) were lower in patients who developed postoperative delirium (DEL+) compared with those who did not (DEL−), although these differences did not reach statistical significance. This observation is in line with previous studies showing that reduced circulating BDNF concentrations are associated with neurodegenerative and neuropsychiatric conditions, including Alzheimer’s disease, depression, and schizophrenia, all characterized by impaired synaptic plasticity and neuronal resilience [31,53,54]. These findings also align with Grandi et al., who reported that reductions in BDNF preceded delirium onset [55]. However, we did not observe dynamic fluctuations in BDNF trajectories that could serve as predictive biomarkers. While BDNF remains biologically plausible as a marker of neurocognitive resilience, its clinical utility as a standalone predictor of POD remains uncertain.

It is conceivable that lower perioperative BDNF concentrations may reflect a pre-existing vulnerability of neuronal networks and a reduced capacity for neuroplastic recovery after major surgical stress. The observed trend toward lower BDNF levels in the DEL+ group, even in the absence of statistical significance, may therefore suggest a pathophysiological link between decreased neurotrophic support and the development of delirium. However, as demonstrated by the lack of significant differences in anesthesia duration, CPB time, total surgical time, and transfusion rates, intraoperative factors alone are unlikely to explain these findings. Future studies with larger cohorts and serial assessments of BDNF kinetics are warranted to clarify whether perioperative BDNF decline represents a contributing mechanism or merely an epiphenomenon in delirium pathogenesis.

### 4.6. Val66Met Polymorphism

The Val66Met polymorphism, which affects activity-dependent BDNF secretion, has been implicated in susceptibility to neurodegenerative and psychiatric disorders [27,32,56]. In our population, the mutation frequency was 31.8%, consistent with prior studies in Poland and comparable to frequencies reported in other Caucasian cohorts [28]. Unexpectedly, carriers of the Val66Met variant exhibited significantly higher BDNF plasma levels at all time points compared to non-carriers, contradicting prior reports that link the polymorphism to impaired BDNF release and reduced synaptic plasticity [27,28,32]. Importantly, the presence of the polymorphism was not associated with delirium incidence or with changes in BDNF kinetics, suggesting that its influence may be context-dependent or modulated by additional genetic and environmental factors.

### 4.7. Limitations

This study has several limitations. The most significant is the relatively small sample size, largely attributable to pandemic-related restrictions on elective CABG procedures and stringent inclusion criteria aimed at ensuring a homogeneous cohort. While these restrictions enhance internal validity, they limit generalizability. Furthermore, intraoperative exclusions due to surgical scope changes further reduced the analytic population. Despite these constraints, the study provides valuable insights by focusing exclusively on isolated CABG, in contrast to most previous research that combined CABG with valve procedures, thereby diluting procedure-specific associations. The relatively small sample size limited the statistical power of the regression analysis and the ability to adjust for multiple confounders. As a result, the regression findings should be interpreted cautiously and viewed as exploratory, generating hypotheses for future, larger-scale studies designed to validate these associations.

### 4.8. Implications and Future Directions

Our findings reinforce the multifactorial nature of postoperative delirium, with preoperative frailty markers, renal complications, and neurotrophic regulation all playing potential roles. The unexpected association of the Val66Met polymorphism, common genetic variant of the BDNF gene, with elevated BDNF levels warrants replication in larger cohorts and may suggest compensatory mechanisms in response to surgical stress. The Val66Met polymorphism has been implicated in psychiatric and neurodegenerative conditions. Carriers of the Met allele may be more vulnerable to memory disturbances and neuroinflammatory responses, especially under surgical stress; therefore, our findings suggest that BDNF Val66Met may represent a genetic determinant of susceptibility to postoperative delirium.

Future studies should integrate multimodal biomarkers—including inflammatory mediators, neuroimaging, and genetic profiling—to improve risk stratification and guide preventive strategies. Ultimately, a precision medicine approach will be essential to mitigate the burden of delirium in vulnerable cardiac surgery patients.

## 5. Conclusions

This prospective single-center study of patients undergoing isolated coronary artery bypass grafting (CABG) did not demonstrate a significant association between perioperative changes in BDNF concentrations or presence of the BDNF Val66Met gene mutation and the incidence of postoperative delirium. The occurrence of postoperative delirium was, however, significantly associated with a prolonged length of hospital stay, although it did not increase the risk of mortality. Neurological complications in the postoperative period, including postoperative delirium, remain an important adverse outcome following cardiac surgery and warrant further investigation. Early identification and management of these disturbances may meaningfully influence patient outcomes and functional recovery after surgery.

## Figures and Tables

**Table 1:** Baseline characteristics of the study cohort (*n* = 107).

Variable	Mean	Me	Q1	Q3
Age (years)	67.01	67.0	62.0	71.0
Body weight (kg)	83.91	84.0	74.0	94.0
Height (cm)	168.70	169.0	164.0	175.0
BMI (kg/m^2^)	29.44	29.4	26.6	32.3
Clinical Frailty Scale (CFS)	3.91	4.0	4.0	4.0
EuroSCORE II (%)	4.44	2.4	1.6	4.1
Left ventricular ejection fraction (LVEF, %)	47.28	45.0	40.0	55.0

Legend: Me—median; Q1—first quartile; Q3—third quartile; CFS—Clinical Frailty Scale; BMI—Body Mass Index; LV EF%—left ventricular ejection fraction.

**Table 2:** Comorbidities and medications of the study cohort (*n* = 107).

		Study Cohort (*n* = 107)	
Variable		*n*	%
Sex	Female	18	16.82%
	Male	89	82.56%
Smoking status	Never	30	28.04%
	Current	19	17.76%
	Former (>30 days)	58	54.21%
NYHA scale	1	17	15.89%
	2	64	59.81%
	3	24	22.43%
	4	2	1.87%
CCS scale	1	1	0.93%
	2	30	28.04%
	3	64	59.81%
	4	12	11.21%
Comorbidities			
Hypertension		99	92.52%
Diabetes mellitus		30	28.04%
Hypercholesterolemia		81	75.70%
Previous myocardial infarction		65	60.75%
Peripheral vascular disease		15	14.02%
Transient ischemic attack		5	4.67%
Previous ischemic stroke		14	13.08%
Chronic kidney disease		2	1.87%
Atrial fibrillation		16	14.95%
COPD		5	4.67%
Asthma		5	4.67%
Peptic ulcer disease		13	12.15%
Autoimmune disease		4	3.74%
Malignancy		11	10.28%
Lymphoma/leukaemia		3	2.80%
Thyroid disease		11	10.28%
Medication			
Aspirin (prophylactic dose)		83	77.57%
ADP receptor inhibitors		18	16.82%
Vitamin K antagonists		2	1.87%
NOACs		5	4.67%
Low-molecular-weight heparins		6	5.61%
β-blockers		72	67.29%
ACE inhibitors		84	78.50%
Calcium channel blockers		48	44.86%
Statins		88	82.24%
Nitrates		6	5.61%
Diuretics		26	24.30%
Endothelin/PDE inhibitors		2	1.87%
Oral antidiabetics		24	22.43%
Insulin		9	8.41%

Legend: CCS—Canadian Cardiovascular Society grading of angina pectoris; ADP inhibitor—adenosine diphosphate receptor inhibitor (antiplatelet agent); NOACs—novel oral anticoagulants; ACE inhibitors—angiotensin-converting enzyme inhibitors.

**Table 3:** Baseline characteristics of the study population stratified by subgroups.

Variable	DEL− (*n* = 86)			DEL+ (*n* = 21)			*p*
	Median	Q1	Q3	Median	Q1	Q3	
Age (years)	67.0	61.0	71.0	69.0	65.0	72.0	0.031
Body weight (kg)	83.0	73.0	94.0	86.0	82.0	89.0	0.408
Height (cm)	169.0	162.0	174.0	172.0	165.0	175.0	0.209
BMI (kg/m^2^)	29.6	26.5	32.3	29.1	27.8	32.2	0.781
Alcohol units per day	2.0	1.0	4.0	2.0	2.0	4.0	0.732
EuroSCORE II (%)	2.3	1.5	4.0	3.5	2.4	6.5	0.006
LV EF [%]	45.0	40.0	55.0	45.0	45.0	50.0	0.624
ASA scale	3.0	3.0	3.0	3.0	3.0	4.0	0.257
CFS	4.0	4.0	4.0	4.0	3.0	4.0	0.832

Legend: CFS—Clinical Frailty Scale; BMI—Body Mass Index; ASA—American Society of Anesthesiologists physical status classification; LV EF%—left ventricular ejection fraction.

**Table 4:** Baseline characteristics of the study population–qualitative data.

		DEL− (*n* = 86)		DEL+ (*n* = 21)		*p*
		*n*	%	*n*	%	
Sex	Female	15	17.44%	3	14.29%	0.983
	Male	71	82.56%	18	85.71%	
Education	None	1	1.16%	1	4.76%	0.256
	Primary	12	13.95%	3	14.29%	
	Vocational	34	39.53%	6	28.57%	
	Secondary	32	37.21%	7	33.33%	
	Higher	7	8.14%	3	14.29%	
	Postgraduate	0	0.00%	1	4.76%	
Smoking status	Never	22	25.58%	8	38.10%	0.255
	Current	14	16.28%	5	23.81%	
	Former (>30 days)	50	58.14%	8	38.10%	
Alcohol consumption	No	33	38.37%	6	28.57%	0.558
	Yes (regular)	7	8.14%	3	14.29%	
	Occasional	46	53.49%	12	57.14%	
NYHA scale	1	16	18.60%	1	4.76%	0.144
	2	52	60.47%	12	57.14%	
	3	16	18.60%	8	38.10%	
	4	2	2.33%	0	0.00%	
CCS scale	1	0	0.00%	1	4.76%	0.006
	2	26	30.23%	4	19.05%	
	3	54	62.79%	10	47.62%	
	4	6	6.98%	6	28.57%	
Comorbidities						
		*n*	%	*n*	%	*p*
Hypertension		80	93.02%	19	90.48%	0.691
Diabetes mellitus		24	27.91%	6	28.57%	0.834
Hypercholesterolemia		65	75.58%	16	76.19%	0.822
Previous myocardial infarction		52	60.47%	13	61.90%	0.898
Peripheral vascular disease		9	10.47%	6	28.57%	0.073
Transient ischemic attack		5	5.81%	0	0.00%	0.579
Previous ischemic stroke		8	9.30%	6	28.57%	0.047
Chronic kidney disease		0	0.00%	2	9.52%	0.046
Atrial fibrillation		13	15.12%	3	14.29%	0.806
COPD		4	4.65%	1	4.76%	0.579
Asthma		5	5.81%	0	0.00%	0.579
Peptic ulcer disease		12	13.95%	1	4.76%	0.433
Autoimmune disease		4	4.65%	0	0.00%	0.715
Malignancy		8	9.30%	3	14.29%	0.785
Lymphoma/leukaemia		2	2.33%	1	4.76%	0.895
Thyroid disease		10	11.63%	1	4.76%	0.597
Medication						
Aspirin (prophylactic dose)		67	77.91%	16	76.19%	0.902
ADP receptor inhibitors		16	18.60%	2	9.52%	0.501
Vitamin K antagonists		2	2.33%	0	0.00%	0.847
NOACs		4	4.65%	1	4.76%	0.579
Low-molecular-weight heparins		1	1.16%	5	23.81%	<0.001
β-blockers		61	70.93%	11	52.38%	0.172
ACE inhibitors		70	81.40%	14	66.67%	0.239
Calcium channel blockers		38	44.19%	10	47.62%	0.969
Statins		70	81.40%	18	85.71%	0.884
Nitrates		4	4.65%	2	9.52%	0.733
Diuretics		19	22.09%	7	33.33%	0.429
Endothelin/PDE inhibitors		2	2.33%	0	0.00%	0.847
Oral antidiabetics		19	22.09%	5	23.81%	0.902
Insulin		6	6.98%	3	14.29%	0.519

Legend: ADP inhibitor—adenosine diphosphate receptor inhibitor; an inhibitor of ADP-induced platelet aggregation; NOAC—non–vitamin K antagonist oral anticoagulants; ACEI—angiotensin-converting enzyme inhibitors; Ca-channel blockers—calcium channel blockers.

**Table 5:** Preoperative laboratory testing.

Variable	DEL− (*n* = 86)			DEL+ (*n* = 21)			*p*
	Median	Q1	Q3	Median	Q1	Q3	
Leukocytes [G/L]	7.3	6.2	8.2	6.5	5.6	6.8	0.050
Lymphocytes [G/L]	1.8	1.4	2.3	1.8	1.4	2.2	0.578
Neutrophils [G/L]	4.8	3.6	5.6	3.7	2.9	4.6	0.032
Erythrocytes [G/L]	4.6	4.2	4.9	4.6	4.2	4.7	0.367
Haemoglobin [mmol/L]	8.7	8.2	9.1	8.6	8.3	9.2	0.829
Platelets [G/L]	222.0	193.0	267.0	239.0	188.0	274.0	0.978
Creatinine [mg/dL]	0.9	0.9	1.1	1.1	0.8	1.3	0.193
Urea [mg/dL]	36.6	30.6	41.8	33.5	31.9	52.0	0.754
Glycated haemoglobin (HbA1c) [%]	6.1	5.8	6.8	5.8	5.6	6.2	0.100
C-reactive protein (CRP) [mg/L]	1.1	0.6	2.7	1.4	0.9	5.4	0.100
CK-MB [U/L]	16.5	14.0	20.0	17.0	12.0	21.0	0.851

Legend: CK-MB—cardiac isoenzyme of creatine kinase.

**Table 6:** Intraoperative data.

Variable	DEL− (*n* = 86)			DEL+ (*n* = 21)			*p*
	Median	Q1	Q3	Median	Q1	Q3	
Anesthesia time [min]	182.5	157.0	220.0	185.0	158.0	205.0	0.683
Surgery time [min]	155.0	135.0	190.0	155.0	130.0	185.0	0.793
Cardiopulmonary bypass time [min]	57.5	48.0	71.0	66.0	47.0	76.0	0.388
PRBC transfusion [units]	2.0	2.0	3.0	4.0	2.0	6.0	0.052
FFP transfusion [units]	3.0	3.0	3.0	3.0	3.0	6.0	0.846
Platelet transfusion [units]	5.0	5.0	5.0	5.0	5.0	10.0	0.374
BDNF 0 [pg/mL]	131.9	76.7	276.6	89.9	67.8	124.5	0.094
BDNF 1 [pg/mL]	121.4	72.1	279.7	87.3	65.6	133.1	0.100
BDNF 2 [pg/mL]	148.9	81.3	318.4	125.2	72.1	160.2	0.232
ΔBDNF 2 vs. 0	2.1	−32.2	74.1	8.3	0.3	25.5	0.317
ΔBDNF 2 vs. 1	6.1	−27.8	48.3	2.9	−18.3	57.7	0.903
ΔBDNF 1 vs. 0	0.2	−38.5	40.4	0.0	−16.3	34.9	0.899
		**DEL− (*n* = 86)**			**DEL+ (*n* = 21)**		***p***
Crystalloid cardioplegia		61	70.93%		10	47.62%	0.076
Blood cardioplegia		25	29.07%		11	52.38%	0.077

Legend: Q1—lower quartile; Q3—upper quartile; FFP—fresh frozen plasma; RBC concentrate—red blood cell concentrate; PC—platelet concentrate. BDNF 0—before surgery; BDNF 1—after induction of anesthesia; BDNF 2—after completion of CPB.

**Table 7:** Postoperative data.

	DEL− (*n* = 86)			DEL+ (*n* = 21)			*p*
	Median	Lower	Upper	Median	Lower	Upper	
HLOS [days]	6.00	5.00	7.00	8.00	6.00	9.00	0.012
	**DEL− (*n* = 86)**			**DEL+ (*n* = 21)**			***p***
Reoperation	3	3.49%		4	19.05%		0.036
CRRT	1	1.16%		5	23.81%		<0.001
Mortality	0	0.00%		1	4.76%		0.442

Legend: HLOS—Hospital Length of Stay; CRRT—Continuous renal replacement therapy.

**Table 8:** Cognitive function assessment.

Variable	DEL− (*n* = 86)			DEL+ (*n* = 21)			*p*
	Median	Lower	Upper	Median	Lower	Upper	
MoCA-0	0.00	0.00	1.00	0.00	0.00	1.00	0.257
MoCA-1	1.00	0.00	1.00	2.00	1.00	2.00	<0.001
MoCA-2	0.00	0.00	1.00	1.00	0.00	1.00	0.110
MoCA-3	0.00	0.00	1.00	1.00	0.00	1.00	0.247
RASS Day 1	0.00	0.00	0.00	0.00	0.00	0.00	0.299
RASS Day 2	0.00	0.00	0.00	0.00	−2.00	2.00	0.288
RASS Day 3	0.00	0.00	0.00	0.00	0.00	0.00	0.351
RASS Day 4	0.00	0.00	0.00	0.00	0.00	0.00	0.739

Legend: MoCA—Montreal Cognitive Assessment; RASS—Richmond Agitation-Sedation Scale; MoCA 0—preoperative; MoCA 1—48 h postoperatively; MoCA 2—30 days postoperatively; MoCA 3—1 year postoperatively.

**Table 9:** Genotyping for the BDNF Val66Met polymorphism.

			DEL− (*n* = 86)		DEL+ (*n* = 21)		*p*
Mutation	NO		56	65.12%	17	80.95%	0.126
	YES		30	34.88%	4	19.05%	
Type of mutation	Heterozygous		27	90.00%	4	100.0%	0.782
	Homozygous		3	10.00%	0	0.00%	
Variable	**No mutation (*n* = 73)**			**Mutation (*n* = 34)**			
	Median	Lower	Upper	Median	Lower	Upper	***p***
BDNF 0 [pg/mL]	104.73	68.25	195.52	166.48	81.56	485.35	0.003
BDNF 1 [pg/mL]	101.56	70.91	211.45	163.68	87.02	554.36	0.024
BDNF 2 [pg/mL]	110.24	77.99	226.84	244.89	87.78	638.19	0.010
ΔBDNF 2 vs. 0	2.01	−22.97	33.57	12.81	−48.69	140.54	0.409
ΔBDNF 2 vs. 1	6.03	−20.31	40.81	6.92	−45.31	154.26	0.771
ΔBDNF 1 vs. 0	0.00	−29.98	34.49	−0.50	−48.89	109.94	0.955

Legend: BDNF 0—before surgery; BDNF 1—after induction of anesthesia; BDNF 2—after completion of CPB.

**Table 10:** Multivariable logistic regression.

Variable	*p*	Odds Ratio	OR 95% CI Lower	OR 95% CI Upper
Val66Met Mutation	0.170	0.439	0.135	1.424
BDNF 0	0.326	0.999	0.998	1.001
BDNF 1	0.241	1.000	1.000	1.000
BDNF 2	0.244	1.000	1.000	1.000
Hospital length of stay (HLOS) [days]	0.009	1.204	1.048	1.383
Reoperation	0.021	6.510	1.334	31.773
Continuous renal replacement therapy (CRRT)	0.004	26.563	2.907	242.746
Leukocytes [G/L]	0.198	0.824	0.614	1.106
Lymphocytes [G/L]	0.521	0.803	0.412	1.568
Neutrophils [G/L]	0.105	0.731	0.501	1.067
Erythrocytes [G/L]	0.149	0.499	0.194	1.284
Haemoglobin [mmol/L]	0.755	0.919	0.539	1.564
Platelets [G/L]	0.611	0.998	0.991	1.005
Creatinine [mg/dL]	0.080	5.336	0.821	34.682
Urea [mg/dL]	0.541	1.003	0.993	1.013
Glycated haemoglobin (HbA1c) [%]	0.264	0.680	0.346	1.338
C-reactive protein (CRP) [mg/L]	0.039	1.187	1.008	1.397
CK-MB 0	0.564	1.013	0.969	1.059
CK-MB 1	0.746	1.004	0.981	1.028
CK-MB 2	0.786	1.003	0.984	1.022
CK-MB 3	0.663	1.004	0.985	1.024
Age ≥ 65	0.186	2.092	0.701	6.244

Legend: BDNF 0—before the start of surgery; BDNF 1—after anesthesia induction; BDNF 2—after completion of cardiopulmonary bypass (CPB); HLOS—Hospital Length of Stay; CK-MB—creatine kinase-MB cardiac isoenzyme.

## Data Availability

Data supporting the findings of this study can be obtained from the corresponding author upon reasonable request by researchers.
